# In-Ear EEG Based Attention State Classification Using Echo State Network

**DOI:** 10.3390/brainsci10060321

**Published:** 2020-05-26

**Authors:** Dong-Hwa Jeong, Jaeseung Jeong

**Affiliations:** 1 Department of Bio and Brain Engineering, College of Engineering, Korea Advanced Institute of Science and Technology (KAIST), Daejeon 34141, Korea; donghwa@kaist.ac.kr; 2 Program of Brain and Cognitive Engineering, Korea Advanced Institute of Science and Technology (KAIST), Daejeon 34141, Korea

**Keywords:** In-ear EEG, echo state network (ESN), attention monitoring, vigilance task

## Abstract

It is important to maintain attention when carrying out significant daily-life tasks that require high levels of safety and efficiency. Since degradation of attention can sometimes have dire consequences, various brain activity measurement devices such as electroencephalography (EEG) systems have been used to monitor attention states in individuals. However, conventional EEG instruments have limited utility in daily life because they are uncomfortable to wear. Thus, this study was designed to investigate the possibility of discriminating between the attentive and resting states using in-ear EEG signals for potential application via portable, convenient earphone-shaped EEG instruments. We recorded both on-scalp and in-ear EEG signals from 6 subjects in a state of attentiveness during the performance of a visual vigilance task. We have designed and developed in-ear EEG electrodes customized by modelling both the left and right ear canals of the subjects. We use an echo state network (ESN), a powerful type of machine learning algorithm, to discriminate attention states on the basis of in-ear EEGs. We have found that the maximum average accuracy of the ESN method in discriminating between attentive and resting states is approximately 81.16% with optimal network parameters. This study suggests that portable in-ear EEG devices and an ESN can be used to monitor attention states during significant tasks to enhance safety and efficiency.

## 1. Introduction

Humans are placed in many situations where it is necessary to sustain attention, such as working, studying, driving, and exercising. However, it is difficult to maintain rigorous attention for a long time. For instance, when subjects were placed in a laboratory setting, their level of attention immediately dropped within 30 min and gradually decreased further over time [1]. The decrease in attention was accelerated as the workload—and, thus, the cognitive demand—increased [2,3]. Degradation of attention sometimes results in dire consequences, for instance, at construction sites, in cars, at hospitals, or on battlefields. Loss of attention has been reported to have severe consequences such as failure to learn or work [4], medical malpractice [5], and traffic accidents [6]. Thus, it is important to monitor attention states during significant tasks requiring high levels of safety and efficiency, and if the level of attention is reduced during such tasks, it is important to take appropriate actions aimed at preventing critical mistakes and improving performance.

There has been a large body of studies on monitoring attention states through techniques that measure brain activity. Electroencephalography (EEG), which records the summed electrical potential from a large ensemble of neurons beneath electrodes, is the most common method used for attention monitoring because it is more portable and cost-effective than other neuroimaging techniques. The theta (4–8 Hz) and alpha (8–11 Hz) bands within EEG signals are known to be associated with the level of attention during a task [7]. In addition, gamma (>30 Hz) oscillations are regarded as an EEG correlate of sustained attention and high cognitive performance [8]. Measuring these electrophysiological features using portable EEG devices has been used to detect attention states during the performance of tasks. However, conventional headsets or cap-shaped EEG devices are uncomfortable to wear in daily life. On the other hand, as mobile phones have advanced to provide multimedia services, earphones are now an essential accessory for smartphone users. Thus, a novel earphone-shaped instrument measuring EEG signal in the ear canal and around the ears has emerged as a strong candidate among attention-measuring devices.

Since the in-ear EEG concept was first introduced [9], a few groups have reported in-ear EEG device prototypes and their signal-detecting properties [10,11,12,13,14,15,16]. In-ear EEG signals show alpha attenuation, defined as the suppression of alpha activity (at approximately 10 Hz) when subjects open their eyes. In-ear EEG signals are highly correlated with on-scalp EEG signals recorded from electrodes near the temporal regions. EEG characteristics, such as auditory steady-state responses (ASSRs) [9,10,12,13,15,16], event-related potentials (ERPs) [9,10,11,12], steady-state visual evoked potentials (SSVEPs) [9,16,17], and sleep-related EEG signals [18,19,20] have been detected and identified from in-ear EEG signals.

There has been a small body of work using in-ear EEG signals to classify brain states using brain–computer interface (BCI) techniques. Most previous studies using in-ear signals and BCI paradigms are synchronous or reactive systems that use external cues, such as ERPs [11,21,22], ASSRs [23], and SSVEPs [17]. The P300 ERP component, which is elicited by target stimuli, is detected with approximately 85% accuracy [11]. When two different sound stimuli are delivered to the right and left ears, the attended stream can be identified from P300 components with approximately 77% accuracy [12]. SSVEPs, which are elicited by visual stimulation at specific frequencies, can be classified with 79.9% accuracy [17]. Since these paradigms are dependent on external visual or auditory stimuli, they cannot be used to detect mental states that require constant monitoring independent of external stimuli. To our knowledge, only a few studies have reported the use of an asynchronous or active BCI that detects mental states in individuals. One study has reported that drowsiness during driving simulations can be recognized from in-ear EEG signals with approximately 85% accuracy over 10 s epochs and 98.5% over 230 s epochs [24]. A similar study to measure day time drowsiness reported that in-ear EEG signals during 30 s epochs of drowsiness were discriminated from 30 s epochs of wakefulness with 80% accuracy [25]. Another study reported that mental workload and motor action during a visuomotor tracking task were detected using a two-channel in-ear EEG system with 68.55% accuracy in 5 s windows and 78.51% accuracy when a moving average filter was applied over five such windows [26]. One study has reported that in-ear EEG signals could be distinguished when subjects viewed emotional pictures for 30 s [27]. In binary classification tasks, positive valence and negative valence could be discriminated with 71.07% accuracy and high and low arousal could be discriminated with 72.89% accuracy. A four-way classification task using all combinations of high or low valence and high or low arousal group was performed with 54.89% accuracy. These studies successfully detected mental states such as drowsiness, mental workload, and emotional states, but long time windows were required for successful classification. A reduced time window and increased classification performance are necessary for asynchronous BCI systems that monitor mental states for the prevention of attention lapses.

The aim of this study is to examine the possibility of discrimination between the attentive state and the resting state using in-ear EEG signals for the potential development of portable, convenient earphone-shaped EEG instruments for attention monitoring. In this study, we recorded both in-ear and on-scalp EEG signals in the attentive state from 6 subjects during the performance of the visual vigilance task. We have designed and developed in-ear EEG electrodes customized by impressions of both the left and right ear canals of the subjects.

In this study, more importantly, we have used an echo state network (ESN), a branch of reservoir computing which is one of the powerful algorithms in machine learning techniques, to discriminate attention states using in-ear EEG. The recurrent property of reservoirs (internal units) in an ESN has been used to provide powerful prediction of nonlinear time series data [28,29,30,31]. Since EEG signals are highly nonlinear and nonstationary, an ESN has been used for EEG prediction, such as monitoring epileptic seizures [32], distinguishing ERP signals elicited by emotional stimuli [33,34], and decoding the intention to move in different directions [35]. These studies have demonstrated that an ESN is more effective than other EEG feature extraction methods. Additionally, ESNs have distinguished human mental states with higher performance than other machine learning classifiers. Therefore, we hypothesize that ESNs are potentially useful for detecting attention states using in-ear EEG signals.

## 2. Materials and Methods

### 2.1. Data Acquisition

**Figure 1:**
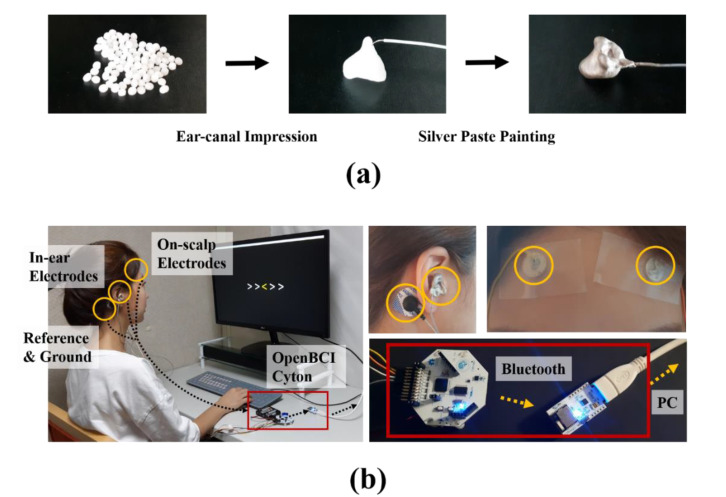
The design of the in-ear electroencephalography (EEG) electrodes. (**a**) Impressions were taken of the ear canal using moldable plastic beads, and conductive silver paste was painted on the impressions for electrical conductivity. (**b**) The participants wore in-ear EEG electrodes on both ears and an on-scalp electrode on either side of the forehead. The mastoid processes were used for the reference and ground channels. Each electrode was connected to an OpenBCI Cyton Board, and then EEG signals were transmitted to a computer (PC) via Bluetooth technology.

In this study, we used moldable plastic beads (InstaMorph, Happy Wire Dog, LLC. USA) and conductive silver paste (ELCOAT P-100, CANS, Japan) to develop in-ear EEG electrodes to place in the ear canal. Ear canal impressions were taken with InstaMorph and connected to electric leads. Then, conductive silver paste was painted on the impressions for electrical conductivity (Figure 1). An in-ear EEG electrodes was placed in each ear. Flat silver disks were produced to place the on-scalp electrodes on the forehead (right and left). Ag/AgCl foam electrodes with conductive adhesive hydrogel (Kendall^®^, Coviden, USA) were used for the ground and reference channels. Lead wires attached to each electrode were connected to an OpenBCI Cyton Board (32 bits, 250 Hz sampling rate). The validity of biosignal acquisition using the developed electrodes was tested and identified by measuring electrocardiography (EKG) signals. The right mastoid process (behind the ear) was selected as the reference site, and the left mastoid process was selected as the ground site. In addition, on-scalp EEG was performed on the forehead (Fp1 and Fp2) under the same conditions as the in-ear EEG to compare the two types of signals.

### 2.2. Participants

Six right-handed participants between 25 and 30 years old were recruited (mean age = 28.17 ± 2.32 years, 4 males) for this study. All participants had normal or corrected vision and no history of neuropsychiatric disease or ear-related problems. We took impressions of participants’ ear canals three days before the experiment. The participants were asked to sleep a sufficient amount and abstain from smoking, alcohol, and caffeine for at least 24 h before the experiment.

Signed consent forms for the experiment were obtained from all participants after the nature of the experiment and the associated precautions had been explained to them. Participants received financial compensation for participating in this experiment, and additional rewards were given based on their task performance. Participants could quit the experiment whenever they felt too tired to maintain their attention. The study and all experimental processes were approved by the institutional review board (IRB) of KAIST.

### 2.3. Experimental Stimuli and Protocol

To verify the in-ear EEG acquisition, we obtained eyes-closed and eyes-open resting-state signals to identify alpha attenuation after cleaning the ear canals with ethanol (the results are shown in Appendix A). Then, attention states were elicited by a visual vigilance task, which was modified from a psychomotor vigilance task (PVT) [36] and the Eriksen flanker task [37]. PVTs are widely used for identifying sustained attention and behavioral alertness by measuring a subject’s reaction time to a specific visual stimulus [38,39]. In general, subjects are asked to press a button as fast as possible when a red dot appears on a monitor. Response-stimulus intervals vary randomly from 2 to 10 s. The Eriksen flanker task is also a widely used task to measure selective attention and executive functions [40,41]. Subjects are asked to press a button corresponding to the target stimulus presented at the center of the screen as quickly as possible, regardless of the flanker stimuli surrounding the target.

**Figure 2:**
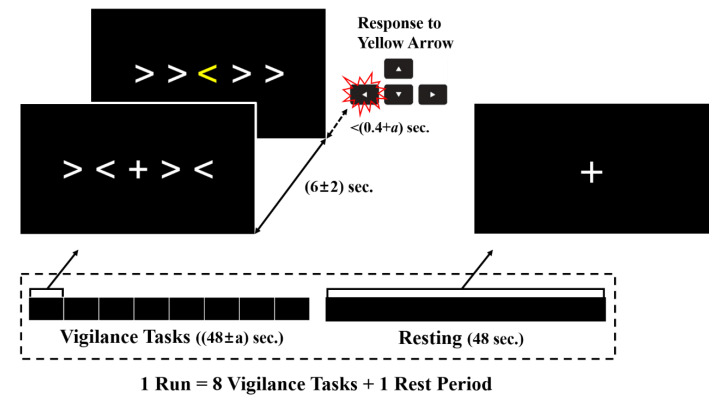
The task for eliciting the attention and resting states. The upper left inset shows the paradigms of the visual vigilance tasks; the target cue centered on the monitor (yellow arrow) was randomly presented with congruent or incongruent flankers. Participants were to press the arrow key corresponding to the target cue as quickly as possible, regardless of the flankers. After 8 trials of vigilance tasks, the participants rested for 48 s while trying not to move.

Since those two tasks are often used for measuring a subject’s attention state, a visual vigilance task combining the two could effectively induce users to maintain their attention with minimal movement during the EEG recording (Figure 2). The participants in this study were asked to focus on a fixation cross centered on a monitor and to press the right or left arrow key when stimuli were presented. The stimuli consisted of five successive arrows pointing in two opposite directions (left or right); one yellow target arrow was positioned at the center, and four white flankers were positioned to the left and right of the target arrow. Two types of flanker arrays were presented: Congruent and incongruent. The congruent flankers pointed in the same direction as the target, and the incongruent flankers pointed the opposite direction from the target. The two flanker types were equal in number and randomly permuted. The time interval from the presentation of the fixation cross to the stimulus in each trial was 6 ± α seconds, where α is a random number less than 2. EEG data collected during this period, when participants were paying attention while expecting to see the stimuli, were regarded as the signal of an attentive state. Moreover, the EEG signal taken during this time would not be corrupted by motion artifacts from keystrokes. If the participants responded before a certain threshold time, they received additional rewards. The threshold time was initially set to 0.4 s in the practice session but was adjusted for each run depending on each participant’s performance to encourage them. Each run consisted of 8 self-paced trials. After one run, the participants rested for 48 s while trying not to move. The resting period of 48 s was set to obtain a dataset of a similar total length to that of the attention state.

There were a total of ten runs, but the participants could quit the experiment if they felt too exhausted to maintain attention. Therefore, the total numbers of runs and trials were different for each subject. On average, each subject performed 8.17 ± 1.72 runs (min = 6 runs, max = 10 runs). The average duration of vigilance trials for each subject was 387.10 ± 83.27 s, and the average resting time was 416.2 ± 89.80 s.

### 2.4. EEG Preprocessing and Feature Extraction

The EEG signals were segmented into windows of 0.5 s (125 points) each and bandpass filtered at 1–50 Hz with a 6th-order Butterworth filter to reduce artifacts. Then, spectral and temporal features were extracted from the filtered signals in epochs of 0.5 s. First, the short-time Fourier transform (STFT) was used to estimate the power spectral densities (PSDs) using an interval of 0.5 s. The square root of the spectral power was subdivided into five EEG frequency bands (*delta*: 1–4 Hz, *theta*: 4–8 Hz, *alpha*: 8–13 Hz, *beta*: 13–30 Hz, and *gamma*: 30–50 Hz). Second, five temporal features for EEG signals corresponding to five EEG frequency bands were also extracted. The EEG signals were filtered with five bandpass filters according to EEG frequency bands (i.e., *delta*, 1–4 Hz; *theta*, 4–8 Hz; *alpha*, 8–13 Hz; *beta*, 13–30 Hz; and *gamma*, 30–50 Hz). The mean amplitude, standard deviation, peak-to-peak amplitude, skewness, and kurtosis were calculated for 0.5 s windows for each frequency band. In total, 10 spectral features (5 frequency bands × 2 channels (right and left)) and 50 temporal features (5 measurements × 5 frequency bands × 2 channels) were collected (Table 1).

All input features were standardized using the following equation:

(1) $$\bar{F_{\mathrm{ch}}}=\frac{F_{ch}-mean\left(F_{ch}\right)}{std\left(F_{ch}\right)}$$

where $F_{ch}$ denotes the original value of an input feature from each channel. Standardized features $\bar{F_{\mathrm{ch}}}$ were also rescaled to a range of −1 to 1, and used as inputs for the classification of resting versus attentive states. The preprocessing and feature extraction were performed with MATLAB Signal Processing Toolbox.

### 2.5. Echo State Network (ESN)

**Figure 3:**
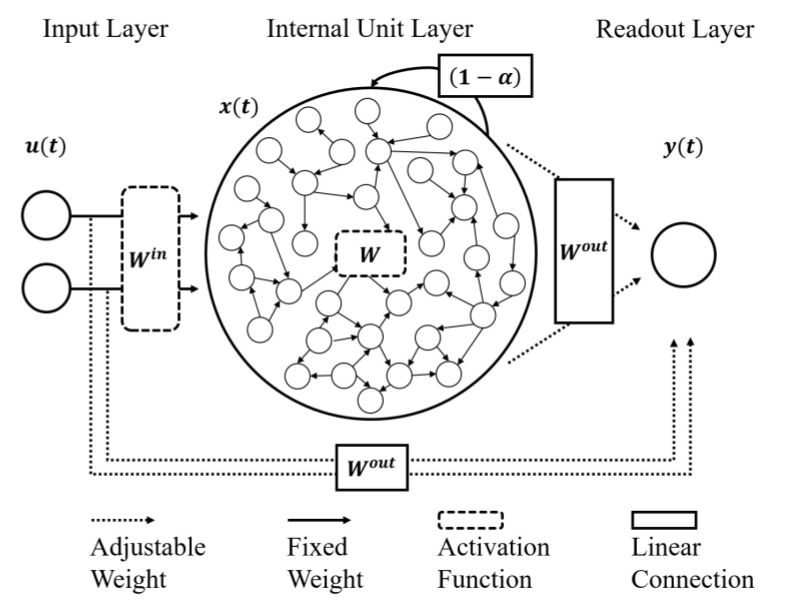
The structure of the echo state network (ESN). The ESN consisted of an input layer (2 input units in this study), an internal unit layer and a readout layer (1 readout). The units of the input layer were connected to the internal units with fixed weights. These internal units were recursively connected to each other with fixed weights. The units of the readout layer were linearly connected from the units of the input and the internal layers with adjustable weights (the figure was modified from [35]).

The discrimination of the attentive and resting states using in-ear and on-scalp EEGs was performed using an ESN. An ESN, which is a type of recurrent neural network (RNN) with a sparsely connected internal unit layer (hidden layer), is recognized as a powerful tool to learn chaotic systems using the recurrent property of biological neural networks [42]. In this study, as presented in Figure 3, the ESN consisted of an input layer, an internal unit layer (also called a reservoir), and a readout layer (also called an output layer). The weights of the neurons in the internal unit layer were initially set to have sparse and random connectivity. The weights of all connections to the readout (output) layer could be tuned to generate specific temporal patterns.

RNNs, including ESNs, have the fading or short-term memory due to the recurrent properties of the internal unit layer. The state of the internal unit, *x*(*t*), is described by the following equation:

(2) $$x\left(t\right)=\left(1-\alpha \right)\cdot x\left(t-1\right)+\alpha \cdot f\left(W^{in}\cdot u\left(t\right)+W\cdot x\left(t-1\right)\right),$$

where *u*(*t*) is an input vector at time step *t* with *W^in^*, the weight matrices between the input and internal units. Vector *x*(*t* − 1) was the previous state of the internal unit with *W*, the weight matrices within internal units. The most distinctive characteristics of ESNs compared to conventional RNNs is that *W* is randomly generated and fixed during learning. Function *f* is the activation function, and *α* is the leaking rate of the reservoir. The hyperbolic tangent (*tanh*) function was used as the activation function in this study. The units of the readout layer *y*(*t*) were updated according to the following equation:

(3) $$y\left(t\right)=W^{out}\left(u\left(t\right),x\left(t\right)\right),$$

where (*u*(*t*),*x*(*t*)) is the concatenation of input and internal units. The feedback from the previous output *y*(*t*) can be delivered to the next internal state *x*(*t* + 1) and output *y*(*t* + 1) but it was not used for this study (for details, see [35]). The echo state, the current state of the internal unit layer, was continuously updated by input streams. The most recent input had the most influence on the echo state, and the influence of any given input decayed over time [43]. Due to this recurrent property of the “reservoir”, ESNs are particularly useful for the prediction of nonlinear, complex time series.

Another characteristic feature of ESNs is that they use simpler learning methods than conventional RNNs. The input layer of an ESN is linearly connected to the internal units (*W^in^*∙*u*(*t*)) and the readout layer (*W^out^*∙*u*(*t*)). The internal units have recursive connections (*W*∙*x*(*t*−1)) and are linearly connected to the readout layers (*W^out^*∙*x*(*t*)). Any linear learning rules can be applied to the ESN because the weights of the input and internal units (*W^in^* and *W*) are randomly selected at the initialization of the network and remain unchanged. Only the weights of the readouts (*W^out^*) were adjusted during linear supervised learning. Despite using a simpler learning rule, ESNs can solve complex problems. Since an ESN has a sufficient number of internal units, the information from the inputs can be expanded to a higher dimension to produce the best solution [44,45,46]. Thus, ESNs have been used in EEG signal analysis [32,33,34,35], brain modeling [47,48,49], and various engineering fields [28,29,30,31].

The selection of parameters is highly significant in constructing an ESN. Many studies on ESNs reported that the spectral radius of the internal weight matrix (*λ*) [50], the leaking rate (*α*) [51,52], the scaling of input weights (*σ*) [53], the size of the internal unit layer (N) [44], and the connectivity (*c*) [45] prominently affected the performance of the those networks. The optimal values of these parameters could vary according to the data.

In this study, the leaking rate and spectral radius were optimized using the grid search method, which created a “grid” of all possible parameters specified by the settings, and calculated the sum of squared errors (SSE) at each one to find the best possible fit. The leaking rate *α* controlled the speed of the reservoir update dynamics. A smaller *α*, which induced the slow dynamics of the reservoir, increased the duration of short-term memory in the ESN [51]. The spectral radius *λ* is the most important feature determining the characteristics of a reservoir. The spectral radius was rescaled to have one as the largest eigenvalue of the internal weight matrix. In theory, a *λ* smaller than one (|*λ_max_*| < 1) was important in the ESN for maintaining the echo state property, i.e., the fading influence of the previous input over time in the reservoir [50]. In practice, however, the spectral radius could be slightly greater than 1, but close to 1 [51,54]. Therefore, in this study, *α* was optimized in the range of (0, 1] and *λ* was optimized in the range of (0, 2]. The step length of the grid search for each parameter was set to 0.1. In total, 200 (10 × 20) ESNs were generated and evaluated for parameter optimization. The ESN with each parameter set was evaluated 10 times. The performances obtained from 10 iterations of grid search were averaged, and the parameters that had the best average performance on average were selected. After the optimization of *α* and *λ* with 100 internal units, the size of the internal unit layer N and the connectivity *c* (sparsity of internal units) were also examined. Although a large reservoir resulted in good performance via regularization to prevent overfitting, it incurred considerable computational costs. Therefore, it was important to find the optimal N. The connectivity *c* was strongly associated with N because it determined the sparsity of the interconnectivity of internal units. Although ESNs were initially designed for sparsely connected reservoirs (1% interconnectivity) to have echo state properties [42], they were reported to work well with fully connected reservoirs [32,52,55]. In this study, the performance of 110 ESNs was evaluated when the number of internal units was 0.1, 0.2, 0.3, …, 1.0, and the connectivity was 0.01, 0.1, 0.2, 0.3, …, 1.0. In addition, 20 ESNs with sparse connectivity (*c* = 0.01, 0.1) were generated for a large reservoir (N = 100, 200, …, 1000).

For the supervised learning of the output weight matrix, Tikhonov regularization (ridge regression) methods were used instead of linear regression, which often leads to numerical instabilities [56]. The regularization parameter was set to a very small value (*β* = 10^−8^) so that the properties would be similar to those of linear regression. Finally, the classification accuracy was obtained with the test set from the optimized and trained ESN. In this study, only one readout was used for the ESN output because there were two classes (resting and attentive states) to distinguish. The attentive states were assigned a value of 1, and the resting states were assigned a value of −1. The predicted states were determined from the values of the readout: the state was classified as an attentive state if the readout returned a positive value or a resting state if the readout returned a negative value.

### 2.6. Data Separation and Evaluation

**Figure 4:**
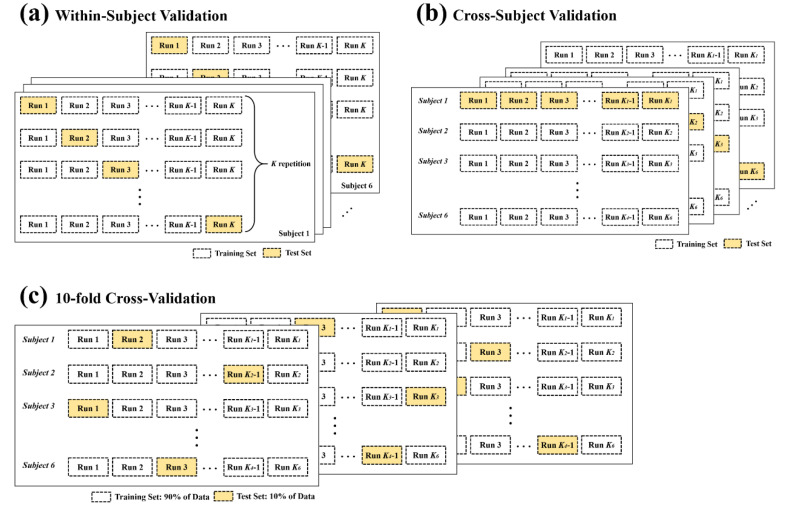
Data separation. (**a**) Within-subject validation was used to train and evaluate the individual classifiers on the attentive and resting states. One run was used for the test set, and the remaining runs were used for the training set. This process was repeated *K* times, and the test set was switched every time. The accuracy was averaged over *K* repetitions. (**b**) Cross-subject testing was performed. The data from one subject were used as a training set, and the data from the other 5 subjects were used as a test set. This process was repeated for each of subjects. (**c**) A generic classifier was evaluated using 10-fold cross-validation. The complete dataset from all subjects was collected and randomly split into a test set (10%) and a training set (90%). This process was repeated 10 times, with a different training set and a different test set each time. The accuracy was averaged over 10 repetitions.

In order to train and evaluate the attention state classifiers, three cross-validation schemes were used. The first cross-validation was within-subject validation, which was designed to evaluate individual classifiers for each subject. The EEG signals were divided into training and test sets based on the total number of runs. When the dataset consisted of *K* runs, *K*−1 runs were used to train the classifier, and the remaining run was used to evaluate the trained classifier. The same process was repeated *K* times by changing the training and test sets as shown Figure 4a. Therefore, classification performances was obtained for each individual subject. Next, cross-subject validation was performed (Figure 4b). The EEG features from one subject were used for testing, and those from the remaining 5 subjects were used for training classifier. This process was repeated for each of 6 subjects. Finally, 10-fold cross-validation was performed to evaluate generic classifiers for all subjects. As presented in Figure 4c, all the data were combined and randomly split into training and test set. For each validation, 90% of data were used for training the classifier, and 10% of data were used for evaluating the trained classifier. This process was repeated 10 times, with a different training and test set each time. In all three cross-validation schemes, attention epochs whose response times were too short (false start < 100 ms) or long (lapse > mean(*RT*) + 3 × std(*RT*)) were not regarded as “attended trials” and were excluded.

## 3. Results

### 3.1. Classification Results

The ESN had a single readout that indicated whether the subject was in an attentive state or a resting state. Because the attentive state was labeled 1 and the resting state was labeled −1, positive readout values were classified as an attentive state, and negative values were classified as a resting state. The classification performance was evaluated using three cross-validation schemes: Within-subject validation, cross-subject validation, and 10-fold cross-validation. Parameter optimization was performed by averaging accuracies obtained from 10 iterations of the grid search. First, individually trained ESN for each subject was evaluated using the within-subject validation. The within-subject validation provided a *K* number of performances if the total number of runs was *K* for each individual. The results of all runs were averaged for each subject. The maximum training accuracy resulting from the grid search was 92.62% on average (Table 2) when in-ear EEG signals were used. The test accuracy using the test set was 81.16%. The results were not much different from those of the on-scalp EEG (82.44%).

**Figure 5:**
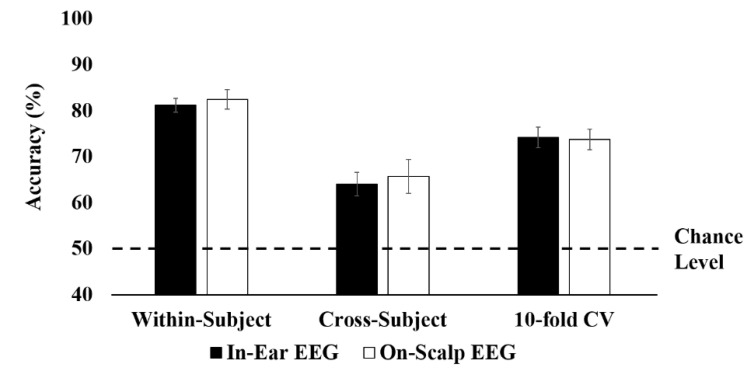
The test accuracy for three cross-validation schemes: within-subject validation, cross-subject validation, and 10-fold cross-validation (CV).

Next, the cross-subject validation and the 10-fold cross-validation were used for evaluating a generic classifier. Table 3 and Figure 5 demonstrated the classification results obtained from two validations. In the 10-fold cross-validation, in which all data were combined and split, the classification accuracy was 74.15% on average when in-ear EEG signals were used (73.73% on average when on-scalp EEG signals were used). These results were slightly lower than those obtained from the within-subject validation, which individually trained and tested for each subject. In addition, cross-subject validation, in which data from one subject were used for test set and data from the other 5 subjects were used for training set, resulted in much lower classification performance (64% for in-ear EEG and 65.7% for on-scalp EEG) than the other two validation schemes.

### 3.2. Smoothing

**Figure 6:**
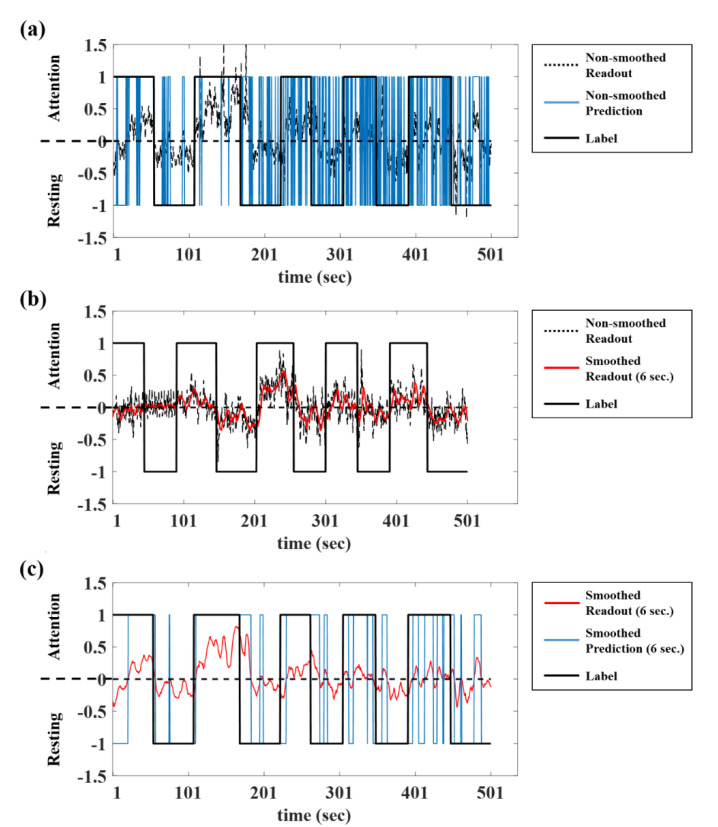
The smoothing of the readout in the ESN. (**a**) The classification results obtained from the original values of the readout fluctuated (black dotted lines: original values of readouts, blue lines: prediction results using original readouts). (**b**) Averaging with previous 6 s outputs corrected the fluctuation (red lines: smoothed readouts using 6 s window). (**c**) The smoothing resulted in improved classification results (blue lines: prediction results using smoothed readouts).

The ESN identified the attentive or resting state in epochs of 0.5 s. The ESN outputs can greatly fluctuate due to the influence of external artifacts or internal states. As seen from the black dotted lines in Figure 6a, the readouts fluctuated with a large amplitude, which leads to rapid fluctuation of predictions (blue lines in Figure 6a).

In order to overcome this problem, the readout values were smoothed using a moving average filter. The current output was the average of itself and n previous outputs when the window size was n as shown below:

(4) $$y\left(t\right)=\sum_{t-n+1}^{t}y\left(t\right),$$

where *y*(*t*) was the current output and *n* was the window size. If there were fewer previous outputs than the window size, the outputs were averaged with every available previous output. The window size was set between 1 and 12 windows (0.5 to 6 s). In Figure 6b, the red lines were outputs smoothed with 6 s windows. The smoothed outputs provided higher classification accuracy than that of the original outputs by reducing fluctuations of readouts (Figure 6c). The average accuracy for the in-ear EEG classification was increased by 2.45% for the within-subject validation, 1.26% for the 10-fold cross validation, and 1.86% for the cross-subject validation (1.03% for the within-subject validation, 0.73% for the 10-fold cross validation, and −0.26% for the cross-subject validation in the on-scalp EEG classification with a 6 s smoothing window). This result indicates that smoothing the readout values successfully reduces their fluctuation and improves the classification performance (Table 4).

### 3.3. Comparison with Conventional Machine Learning Methods

In order to evaluate the discrimination performance of the ESN, various machine learning methods commonly used in EEG classification were also investigated to compare for the in-ear and on-scalp EEG signals. The following 7 machine learning methods were used: (1) Regularized linear discriminant analysis (R-LDA), (2) decision tree (DT), (3) random forest (RF), (4) naïve Bayesian algorithm (NB), (5) k-nearest neighbor algorithm (k-NN), (6) support vector machine (SVM) with linear kernels, and (7) SVM with Gaussian kernels. A detailed explanation of each machine learning methods can be found in Appendix B. The same features used in ESN classification were used for these conventional machine learning methods. The hyperparameters for each classifier were optimized during training. All processes were performed in MATLAB using Statistics and Machine Learning Toolbox. The accuracies obtained from each validation for each conventional machine learning method were compared with those obtained from ESN using Student’s t-test and the multiple comparison problem was corrected using Bonferroni correction.

**Figure 7:**
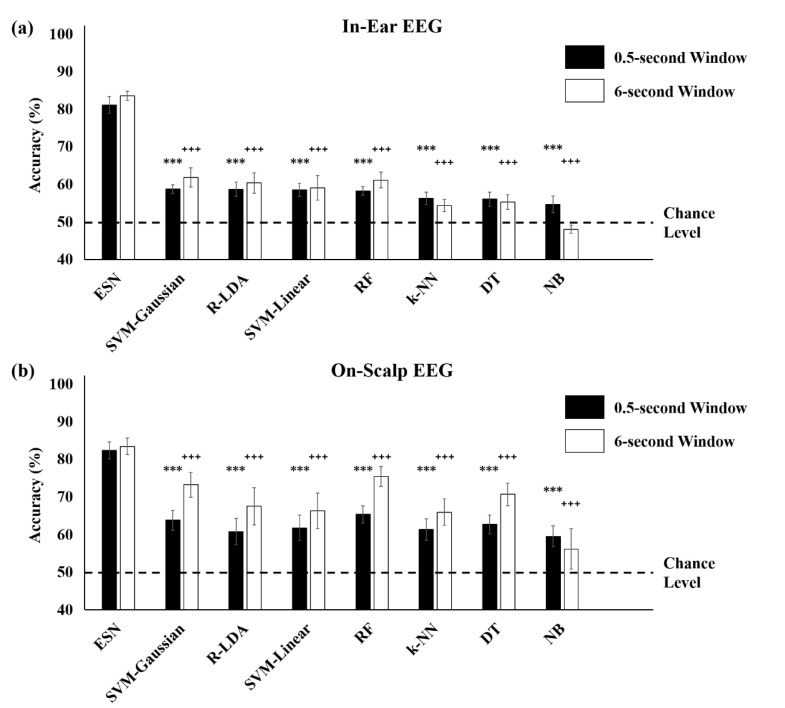
Comparison of classification accuracy between the echo state network (ESN) and conventional machine learning methods and smoothing obtained from the within-subject validation. The ESN classification highly outperformed other conventional machine learning methods in both (**a**) in-ear and (**b**) on-scalp EEGs. The results were sorted in descending order based on the accuracy of nonsmoothed prediction. The dotted line denoted the chancel level (50%). ESN: echo state network, SVM-Gaussian: support vector machine (SVM) with Gaussian kernels, R-LDA: regularized linear discriminant analysis (LDA), SVM-Linear: SVM with linear kernels, RF: random forest, k-NN: k-nearest neighbor algorithm, DT: decision tree, and NB: naïve Bayesian algorithm (*** denotes *p* < 0.001 when comparing original predicted results without smoothing (0.5 s window) of the ESN and other methods, ^+++^ denotes *p* < 0.001 when comparing smoothed results using 6 s window of the ESN and other methods, Bonferroni corrected).

When within-subject validation was conducted (Figure 4a), we found that the ESN resulted in 81.16% for the in-ear EEG (82.44% for on-scalp EEG) classification accuracy without smoothing and 83.62% (83.47% for on-scalp EEG) accuracy after smoothing with a 6 s window. These results significantly outperformed those of the 11 machine learning methods, as shown in Figure 7.

**Figure 8:**
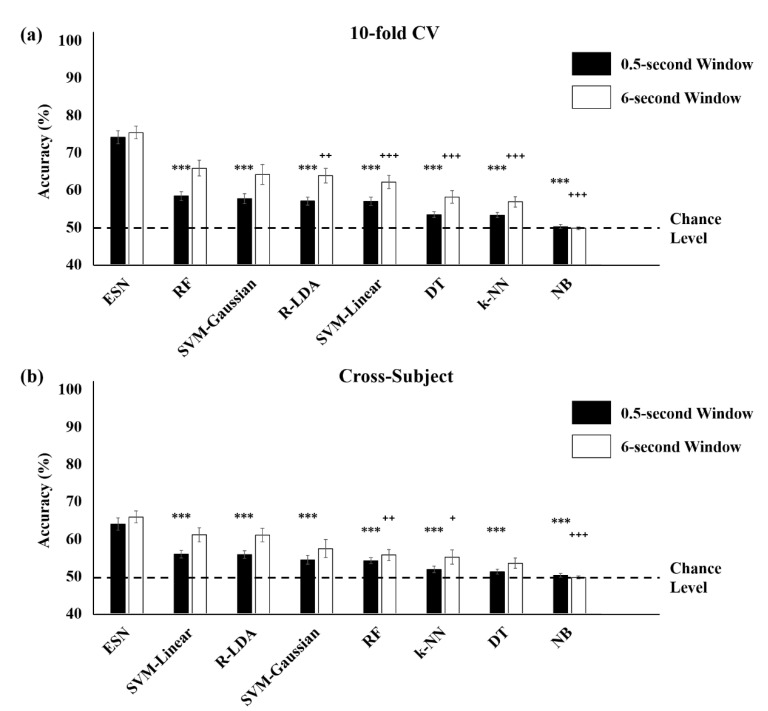
Comparison of classification accuracy between the echo state network (ESN) and conventional machine learning methods obtained from the (**a**) 10-fold cross-validation (CV) and (**b**) cross-subject validation using in-ear EEG signals. The results were sorted in descending order based on the accuracy of nonsmoothed prediction. The dotted denotes the chance level (50%). (*** *p < 0*.001, ** *p* < 0.01, * *p* < 0.05 for comparisons of original predicted results without smoothing (0.5 s window), ^+++^ *p < 0*.001, ^+++^ *p* < 0.01, ^+^ *p* < 0.05 for comparisons of smoothed results using 6 s window, Bonferroni corrected).

The classification results obtained from 10-fold cross validation and cross-subject validation were also higher with an ESN compared to other machine learning methods (Figure 8). The smoothing of classification results with 6 s window led to greater increases in performance in conventional machine learning methods compared to ESN. In the 10-fold cross-validation, the classification accuracies using smoothing classification results obtained from RF and SVM with Gaussian kernels were not significantly different from those that were obtained using the ESN. In the cross-subject validation, SVM with linear kernels, regularized LDA, and SVM with Gaussian kernels provided performances that were statistically not different from those of the ESN. However, the ESN still outperformed these methods for all validations.

## 4. Discussion

It is sometimes critical to maintain attention when carrying out tasks requiring high levels of safety and efficiency in daily life [7,8]. During these tasks, attention monitoring may be helpful for preventing mistakes and improving performance by providing proper solutions, such as neurofeedback or brain stimulation. In this study, we have demonstrated that the ESN classification of in-ear EEG signals is a potentially powerful method to discriminate the attention state from the resting state compared with other conventional machine learning techniques and even with on-scalp EEGs. In addition, we have shown that parameter optimization procedure is important for producing better performance and have suggested the range of optimal parameters in ESN for in-ear EEGs for the highest results.

Based on these results, we suggest that this approach can be applied to the prediction of sleep deprivation and of highly stressful states, as vigilance degradation is associated with lack of sleep [36] and with high levels of anxiety and stress [3,37]. Furthermore, attention monitoring using in-ear EEG and ESNs could potentially aid in the diagnosis of attention-related diseases such as attention deficit hyperactivity disorder (ADHD) [57,58] or Alzheimer’s disease [59,60].

Due to the inconvenience of conventional cap-type or headset-type EEG devices, even though extensive research has been performed, BCI techniques for attention state monitoring have not been widely used in daily life. We suggest that earphone-shaped EEG devices using in-ear EEG signals would be a strong candidate for potential BCI devices in future, which can monitor human mental states including attention states even when the users are listening to music or watching the movies. Since the first research on the “in-the-ear recording concept” was published in 2012 [9], the BCI application of in-ear EEG signals has been investigated using the external stimuli such as visual or auditory cues [11,17,21,22,23] or independently of external stimuli [24,25]. Compared with the performance of the previous studies on the BCI application of in-ear EEG signals to mental state monitoring, our performance using the ESN technique is higher than theirs: Previous studies successfully have detected drowsiness [24,25], mental workload during visuomotor tracking task [26], and emotional states [27] but have required long time window (more than 10 s) to achieve high classification accuracy (Table 5). In this study, we suggest that the attention monitoring system using in-ear EEG and the ESN is much faster to classify mental states than previous studies, within every 0.5 s with high accuracy of 81.16% when using one run as the test set and remaining runs as the training set within each subject. We have demonstrated that the classification accuracy increased to 83.62% after smoothing the classification results with a 6-s window, which is much higher than those of conventional machine learning methods used for EEG classification compared in this study (Figure 7). The classification accuracy was lowered to 74.15% in the 10-fold cross validation, which was performed by combining all features from all subjects and splitting into training and test set, and 64% in the cross-subject validation, which was performed by using data from one subject as test set and data from remaining 5 subjects as training set. However, these results were still outperformed conventional machine learning methods (Figure 8).

The decreased accuracy in the cross-subject validation compared to those in the within-subject model might be resulted from intersubject variability of EEG signals. Because the parameters of ESN greatly affect classification performance, it is important to apply parameter optimization. The optimized parameters obtained from the grid search were varied for each validation. Therefore, in the cross-subject validation, the ESN could not find optimal parameters and thus could not learn distinguishing features for the classification due to the difference of EEG properties for each individual. The spectral radius *λ* and the leaking rate *α* were optimized using the grid-search. The leaking rate, which determines how fast the dynamics of the reservoir are updated, was optimized in the range of (0, 1]. The spectral radius, which determines characteristics of reservoir (short-term or long-term), was optimized in the range of (0, 2]. In theoretical, a *λ* smaller than one was suggested for the echo state property but a *λ* larger than one (but close to 1) can be employed in practice [51,54]. We found that a *λ* larger than one was selected in many cases. Determining the proper size of the reservoir is also important in the performance of ESN. When internal units were sparsely connected to each other, insufficient number of internal units could not extract nonlinear features. Too many internal units resulted in decreased accuracy as well as high computational cost. Although the denser connectivity required higher computational cost, it did not ensure higher accuracy. Therefore, it is important to find the optimal reservoir size and sparsity. The additional discussions about parameter optimization were attached in Appendix C.

Real time prediction of test sets in in-ear EEGs for attention state monitoring may be possible, once the training process is accomplished. However, it is also necessary to train in real time to reduce the computational cost. We should address that the supervised learning method in this study has incurred a high computational cost, even if the size of the dataset is not too large. To monitor mental state continuously in real time, the network needs to be adaptive to new data constantly. Therefore, we will modify and improve the training method suitable for real-time monitoring in future studies.

In this study, we have designed and developed the in-ear EEG electrodes by customizing each subject’s ear canals. It is difficult to develop a generic earpiece that suits all users because the shape and length of both left and right ear canals in each user are different [61]. Therefore, we suggest that generic and more comfortable in-ear electrodes, which can be made flexible with carbon nanotube polydimethylsiloxane (CNT/PDMS) [10] or memory foam substrate [13], are required for the production of earphone-shaped EEG devices suitable for individuals to achieve better measurement performance.

In this study, we have identified only binary mental states: Attention and resting states. The attention states will be further divided into various types of attention states and levels beyond binary classification for our future investigation. In addition, we suggest that this methodology can be potentially expanded to apply to monitoring of other mental states, such as stressfulness, drowsiness and sleepiness, or emotion (positive/negative valence). We also suggest that the ESN and other machine learning techniques are likely useful for analysis of the in-ear EEG signals for mental state monitoring systems. Furthermore, we speculate that earphone-shaped mental state monitoring system using in-ear EEG signals can be a strong candidate device for massive commercial services of BCI.

## 5. Conclusions

This study suggests that the attention state can be detected with high accuracy using the ESN and in-ear EEG signals. The attention states can be discriminated from the resting state for every 0.5 s with 81.16% accuracy when ESN was trained and tested using in-ear EEG signals within each subject. We suggest that this method can be likely applied to asynchronous or active BCIs, which can detect mental states without external stimuli. Unlike synchronous or passive BCIs which use external stimuli, asynchronous or active BCIs are potentially useful in daily life. The smoothing of ESN readouts will be useful for stable BCI systems because large fluctuations of classification results can cause negative effects in practice such as excessive feedback to users. The application of this technology using earphone-shaped EEG devices and the ESN may pave the way for comfortable mental monitoring devices in the near future.

## Figures and Tables

**Table 1:** Description of extracted features.

EEG Bands	Freq. Range	Spectral Feature	Temporal Features				
*Delta*	1–4 Hz	*Delta* power	Mean amplitude	Standard Deviation	Peak to Peak	Skewness	Kurtosis
*Theta*	4–8 Hz	*Theta* power	Mean amplitude	Standard Deviation	Peak to Peak	Skewness	Kurtosis
*Alpha*	8–13 Hz	*Alpha* power	Mean amplitude	Standard Deviation	Peak to Peak	Skewness	Kurtosis
*Beta*	13–30 Hz	*Beta* power	Mean amplitude	Standard Deviation	Peak to Peak	Skewness	Kurtosis
*Gamma*	30–50 Hz	*Gamma* power	Mean amplitude	Standard Deviation	Peak to Peak	Skewness	Kurtosis
Total number of features (in single channel)		5	25				

**Table 2:** The maximum training accuracy and test accuracy for each subject in the within-subject validation.

	In-Ear EEG		On-Scalp EEG	
Subject #	Training Accuracy (%)	Test Accuracy (%)	Training Accuracy (%)	Test Accuracy (%)
1 (M)	92.77 ± 2.14	81.42 ± 4.18	98.14 ± 0.54	91.23 ± 2.13
2 (M)	91.59 ± 1.49	75.89 ± 2.91	88.25 ± 2.26	78.52 ± 2.84
3 (M)	94.11 ± 1.34	83.28 ± 2.88	92.06 ± 1.64	80.64 ± 2.79
4 (M)	93.97 ± 1.76	79.34 ± 3.39	96.25 ± 0.67	85.73 ± 3.82
5 (F)	91.25 ± 3.47	79.46 ± 5.01	92.24 ± 1.07	83.05 ± 4.47
6 (F)	92.00 ± 2.09	87.59 ± 3.25	90.74 ± 1.45	75.46 ± 3.40
Avg.	92.62 ± 0.45	81.16 ± 2.20	92.95 ± 2.19	82.44 ± 2.24

**Table 3:** The maximum training accuracy and test accuracy in the cross-subject validation and 10-fold cross-validation.

		In-Ear EEG		On-Scalp EEG	
Validation Method	Subject #	Training Accuracy (%)	Test Accuracy (%)	Training Accuracy (%)	Test Accuracy (%)
**Cross-Subject Validation**	1 (M)	73.46	62.65	77.17	79.54
	2 (M)	63.55	58.36	74.56	60.12
	3 (M)	66.00	54.59	61.46	55.31
	4 (M)	81.16	65.39	83.33	76.35
	5 (F)	83.55	69.52	72.88	58.63
	6 (F)	85.08	73.47	78.73	64.24
	Avg.	75.46 ± 3.44	64.00 ± 2.60	74.69 ± 2.77	65.70 ± 3.71
**10-fold Cross-Validation**	Avg.	80.89 ± 1.93	74.15 ± 2.20	78.42 ± 2.19	73.73 ± 2.24

**Table 4:** The performances according to smoothing window.

	Within-Subject		Cross-Subject		10-fold CV	
Smoothing Window (second)	In-Ear EEG (%)	On-Scalp EEG (%)	In-Ear EEG (%)	On-Scalp EEG (%)	In-Ear EEG (%)	On-Scalp EEG (%)
Non (0.5 s)	81.16	82.44	64.00	65.70	74.15	73.73
2 (1 s)	81.32	82.73	64.64	65.78	74.75	73.63
4 (2 s)	82.33	83.56	65.86	65.73	75.07	73.89
6 (3 s)	82.90	83.74	65.84	65.64	75.32	74.01
8 (4 s)	83.24	83.81	66.09	65.36	75.36	74.22
10 (5 s)	83.24	83.70	65.97	65.73	75.34	74.54
12 (6 s)	83.62	83.47	65.85	65.44	75.41	74.46

**Table 5:** Comparison with previous studies on asynchronous brain–computer interface (BCI) using in-ear EEG signals (CV denotes cross-validation).

Authors	Mental States (Classes)	Window (Seconds)	Methods	Validation	Accuracy
Hong et al. (2018) [24]	Drowsiness (5 levels)	10	RF	5-fold CV	0.780 (kappa value)
Nakamura et al. (2018) [25]	Drowsiness (Wake vs. N1)	30	SVM	Leave-one trial-out (all subjects)	80%
				10-fold CV (all subjects)	82.9%
Kuatsjah et al. (2019) [26]	Mental workload (Visuomotor task vs. Rest)	5	The best among various ML approaches	Across-trial for each subject	68% (approx.)
		25			79.30%
		5	NN	5-fold CV for each subject	71.50%
Athavipach et al. (2019) [27]	Emotion (Valence)	30	SVM	10-fold CV for each subject	73.01%
	Emotion (Arousal)				75.70%
	Emotion (Valence+Arousal)				59.23%
**This Study**	**Attention (Vigilance task vs. Rest)**	**0.5**	**ESN**	**Across-trial for each subject**	**81.16%**
				**10-fold CV (all subjects)**	**74.15%**
				**Cross-subject**	**64.00%**

The bold types were used for discriminating our results from others’.

**Figure A1:**
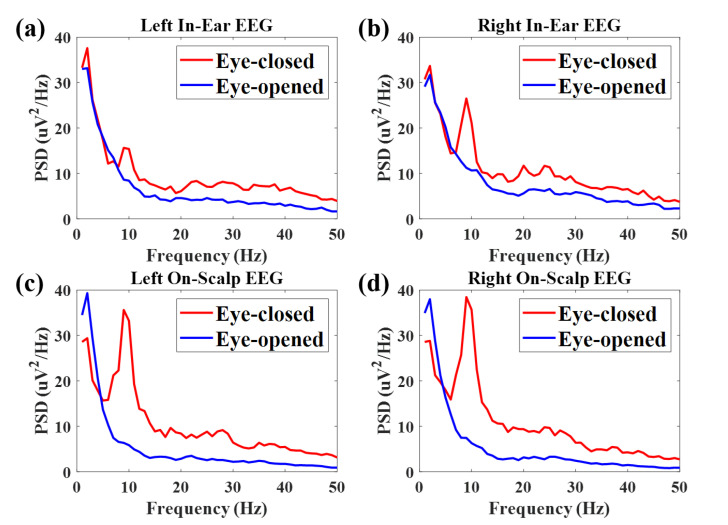
Alpha attenuation effect. Red lines denote averaged power spectral densities (PSDs) during eyes-closed resting state and blue lines are averaged PSDs during eyes-open resting state from (**a**) left in-ear EEG channel, (**b**) right in-ear EEG channel, (**c**) left on-scalp EEG channel, and (**d**) right on-scalp EEG channel.

**Figure A2:**
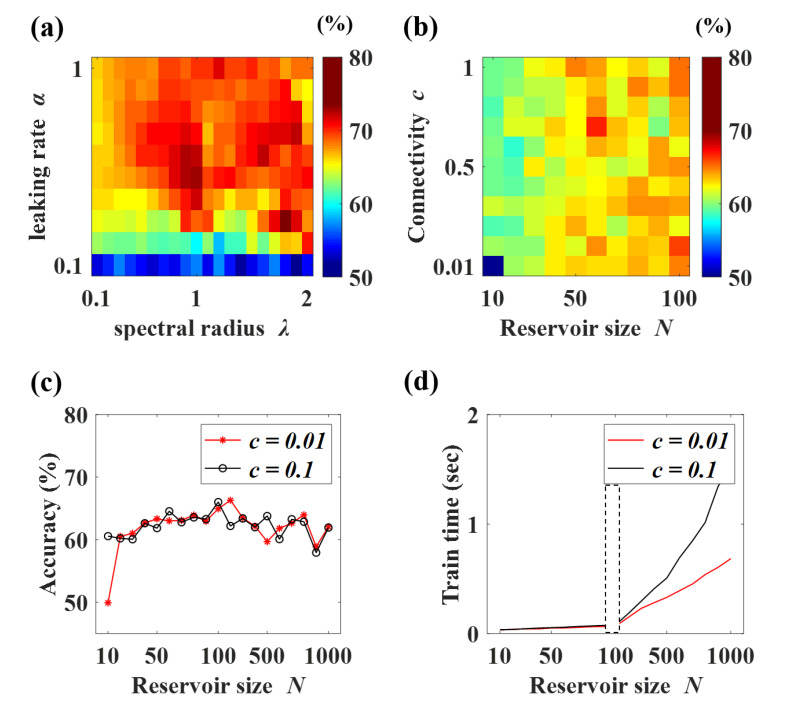
The influences of the parameters in the echo state network (ESN) in the 10-fold cross-validation. (**a**) The average accuracy according to spectral radius *λ* and leaking rate *α*. (**b**) The average accuracy according to the number of internal units (*N* = 10, 20, …, 100) and interconnectivity (*c* = 0.1, 0.2, …, 1). (**c**) Accuracy according to the number of internal units (*N* = 100, 200, …, 1000) with sparse interconnectivity (*c* = 0.1, 0.01). (**d**) The computational cost of the training ESN according to the number of internal units. The computational cost linearly increased as the size increased. The sparser reservoir (*c* = 0.01) had less computational cost compared with the denser reservoir (*c* = 0.1), which became more evident as the reservoir size increased. The dotted box was inserted when the reservoir size was 100 because the N increases in steps of 10 between 10 and 100, and in steps of 100 between 100 and 1000.

## Appendix A

To verify the in-ear and on-scalp EEG acquisition, the alpha attenuation tests were performed prior to the visual vigilance task. It is known that the *alpha* wave shows dominant peak approximately 10 Hz when eyes are closed but decreases when eyes are opened. This phenomenon is widely used to assess EEG signals. The STFT was used to estimate the PSD using a 1-s (250 data points) window with a 50% overlap. The PSDs of each state were averaged and compared for each channel. The alpha attenuation effect was observed both from in-ear and on-scalp EEG signals but the effect was diminished for in-ear EEG signals (Figure A1).

## Appendix B

The classification accuracy of ESN was compared with conventional machine learning methods in Section 3.3. Total 60 features, which is the same feature set used in ESNs (Table 1), were used as features for 7 conventional machine learning methods: (1) R-LDA, (2) DT, (3) RF, (4) NB, (5) k-NN, (6) SVM with linear kernels, and (7) SVM with Gaussian kernels.

Discriminant analysis (DA) is the most widely used classification method, which finds optimal boundaries to separate two or more classes using multivariate observations [62,63]. LDA assumes that observations in all classes have normal distributions with identical covariance. When the covariance matrix is regularized from the observations, it is called (1) regularized LDA (R-LDA). (2) DT learning constructs a predictive model (decision tree) for rule-based classification by splitting multiple binary nodes with input features and pruning trees with labeled outputs [63]. (3) RF constructs multiple DTs in training and classifies the class using multiple outputs from trained trees [64,65,66]. Using multiple DTs reduces overfitting and noise in every tree. (4) NB is a simple probabilistic classifier based on Bayes’ theorem, which infers posterior probability (class) from prior probability, likelihood, and evidence (current observation, i.e., input) [62]. (5) k-NN classifies input observations based on their closeness calculated by Euclidean distance [62]. The classification of new observation (test data) is performed based on the k closest neighbors (training data). The 5-NN was used to avoid overfitting in this study. SVM is a classifier used to find the optimal hyperplane using the largest margin (support vectors) between the two classes [62]. The method for computation of hyperplane is called kernel function. (6) SVM with linear kernel is an originally proposed method for SVM construction, which uses simple inner (dot) products. In the case of nonlinear classification, (7) Gaussian function (SVM with Gaussian kernel) can be used instead of inner products.

## Appendix C

The influence of each parameter was compared using the training results. The optimized parameters (leaking rate *α*, spectral radius *λ*, reservoir size N, and the sparsity of interconnectivity *c*) were varied for each validation. Figure A2a shows the average training accuracy in the 10-fold cross validation when each of the two parameters (*α*, *λ*) was changed. In most cases, the selected *λ* values were larger than 1 or close to 1. The selected *α* values were varied in the rage of 0.3 to 1.

The influences of the reservoir size and connectivity of the internal unit layer were also examined. An insufficient number of internal units (N < 40) resulted in poor classification accuracy, but the accuracy increased as the size increased (Figure A2b,d). ESNs with large reservoir sizes (N = 100, 200, …, 1000) were also generated with sparse connectivity (*c* = 0.01, 0.1). Although the optimal sizes and connectivity were different for each subject, the maximum accuracy was obtained from 200 internal units and 0.01 connectivity. The larger reservoir size had lower classification accuracy in both in-ear and on-scalp EEG. The accuracy was not influenced by sparsity of internal units if the size was large enough. The calculation durations in training ESN were also compared. The calculation time increased linearly as a function of the reservoir size (Figure A2d). We found that the reservoir with lower density (*c* = 0.01) was more computationally effective while maintaining the accuracy compared to the reservoir with higher density (*c* = 0.1). Larger reservoir size increased the gap of computational cost between reservoirs of two different degrees of sparsity.
